# Analytical Solutions Based on Fourier Cosine Series for the Free Vibrations of Functionally Graded Material Rectangular Mindlin Plates

**DOI:** 10.3390/ma13173820

**Published:** 2020-08-29

**Authors:** Chiung-Shiann Huang, S. H. Huang

**Affiliations:** Department of Civil Engineering, National Chiao Tung University, 1001 Ta-Hsueh Rd., Hsinchu 30050, Taiwan; mark071819@gmail.com

**Keywords:** analytical solution, Fourier cosine series, vibrations, FGM rectangular plates, Mindlin plate theory

## Abstract

This study aimed to develop series analytical solutions based on the Mindlin plate theory for the free vibrations of functionally graded material (FGM) rectangular plates. The material properties of FGM rectangular plates are assumed to vary along their thickness, and the volume fractions of the plate constituents are defined by a simple power-law function. The series solutions consist of the Fourier cosine series and auxiliary functions of polynomials. The series solutions were established by satisfying governing equations and boundary conditions in the expanded space of the Fourier cosine series. The proposed solutions were validated through comprehensive convergence studies on the first six vibration frequencies of square plates under four combinations of boundary conditions and through comparison of the obtained convergent results with those in the literature. The convergence studies indicated that the solutions obtained for different modes could converge from the upper or lower bounds to the exact values or in an oscillatory manner. The present solutions were further employed to determine the first six vibration frequencies of FGM rectangular plates with various aspect ratios, thickness-to-width ratios, distributions of material properties and combinations of boundary conditions.

## 1. Introduction

Functionally graded materials (FGMs) were first produced in the mid-1980s [1]. An FGM is composed of varying mixtures of different materials, such as ceramics and metals. The material properties of FGMs smoothly and continuously vary, in contrast to conventional laminated composite materials. Consequently, FGMs do not comprise stress singularities formed due to discontinuities in the material properties. FGMs can be designed to possess the high heat resistance and corrosion resistance of ceramics as well as the high mechanical strength of metals. Over the previous three decades, FGMs have been extensively explored in various fields including aerospace, energy, electronics, optics, biomedicine, and mechanical engineering.

Plates are employed in a wide range of mechanical and structural system components in civil, mechanical and aeronautical engineering. The behaviors of FGM plates have attracted research attention. Different reviews [2,3,4,5,6] have provided exhaustive summaries of the studies published on the free vibrations and buckling of FGM plates according to various plate theories and the three-dimensional elasticity theory.

Numerous studies have investigated the free vibrations of FGM rectangular plates, and most of these studies have employed various numerical methods. For example, on the basis of the classical plate theory, Abrate [7] and Zhang and Zhou [8] reported that an FGM plate behaves similar to a homogeneous plate if a suitable reference plane is adopted, while Yang and Shen [9] employed a one-dimensional differential quadrature approximation and the Galerkin procedure to determine the frequencies of initially stressed plates. According to the first-order shear deformation plate theory, Zhao et al. [10] applied the element-free kp-Ritz method to analyze the vibrations of square and skew plates under different combinations of boundary conditions. Fu et al. [11] employed the Ritz method with admissible functions consisting of double Fourier cosine and several closed-form auxiliary functions to study the vibrations of orthotropic FGM plates with general boundary restraints. Ferreira et al. [12] investigated the vibrations of square plates employing the first-order and third-order shear deformation plate theories and the collocation method with multiquadric radial basis functions. Huang et al. [13] adopted the third-order shear deformation plate theory and Ritz method for analyzing the vibrations of rectangular plates with and without side cracks. Hong [14] investigated the thermal vibrations of plates via the generalized differential quadrature method and the third-order shear deformation plate theory. Using the higher-order shear deformation plate theories, Qian et al. [15] applied the Petrov–Galerkin meshless method and Roque et al. [16] adopted the multiquadric radial basis function method to find the vibration frequencies of thick plates. Using the Ritz method and three-dimensional elasticity theory, Uymaz and Aydogdu [17] studied the vibrations of plates with various combinations of boundary conditions, and Cui et al. [18] performed vibration analysis of an FGM sandwich rectangular plate resting on an elastic foundation using admissible trigonometric functions. Huang and his coworkers [19,20] proposed a set of admissible functions, which can accurately describe the behaviors of a crack, for examining the vibrations of cracked FGM rectangular plates and also showed the natural frequencies of intact plates. Burlayenko et al. [21] employed the commercial finite element package ABAQUS to analyze the vibrations of thermally loaded FGM sandwich plates.

Only a few studies have been devoted to analytical solutions for the free vibrations of FGM rectangular plates based on various plate theories. The solutions in these studies consider rectangular plates with two opposite edges or four simply supported edges (faces). Using the first-order shear deformation plate theory, Hosseini-Hashemi et al. [22,23] introduced new potential and auxiliary functions to construct exact closed-form solutions for the vibrations of rectangular plates having two opposite edges simply supported with and without considering the in-plane displacement components, respectively, while Ghashochi-Bargh and Razavi [24] proposed analytical solutions for the vibrations of orthotropic FGM rectangular plates without considering the in-plane displacement components. Hosseini-Hashemi et al. [25] extended their studies by using a third-order shear deformation theory. Considering simply supported conditions on four edge surfaces, Matsunaga [26] and Sekkal et al. [27] developed solutions for FGM rectangular plates and sandwich plates, respectively, according to the higher-order shear deformation plate theories. Based on three-dimensional elasticity theory, Vel and Batra [28] used the power series method to construct solutions for the vibrations of FGM rectangular plates, while Reddy and Cheng [29] employed an asymptotic approach along with a transfer matrix. Huo et al. [30] employed the recursive matrix method to develop the solutions for the vibrations of FGM sandwich plates.

According to plate theories, there are 21 distinct combinations of boundary conditions (i.e., free, simply supported and clamped) for rectangular plates. The literature review found that except for the six cases in which two opposite edges are simply supported, no analytical solution for the vibrations of FGM rectangular plates with various combinations of boundary conditions exists. The present study aims to fill a gap in the literature and proposes analytical solutions based on the Mindlin plate theory for the vibrations of FGM rectangular plates with 21 combinations of boundary conditions. The proposed solutions are established using the Fourier cosine series with polynomial supplementary functions, which eliminate the validity requirement for the term-wise differentiation of the Fourier sine series of a function to accurately represent the differential of the function [31]. The validity of the present solutions is confirmed through comprehensive convergence studies for plates with four combinations of boundary conditions and by comparing the obtained vibration frequencies with those published in the literature. The material properties along the thickness of an FGM plate are estimated using the power-law or the Mori–Tanaka scheme. The solutions are further applied to determine the vibration frequencies of Al/Al_2_O_3_ FGM plates with nine combinations of boundary conditions. The obtained analytical results can serve as a benchmark for the evaluation of other solutions obtained through various approximate or numerical approaches.

## 2. Methodology

### 2.1. Material Models

**Figure 1:**
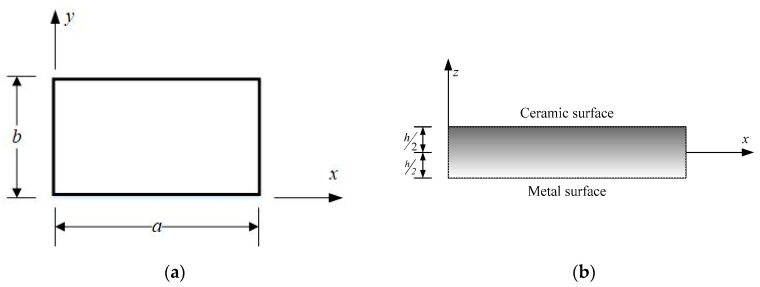
Geometry of a functionally graded material (FGM) plate and coordinates: (**a**) top view, (**b**) side view.

Depicted in Figure 1 is a rectangular FGM plate with a length of *a*, width of *b* and thickness of *h*. The material properties of FGM plates are assumed to vary along their thickness (*z*) according to the power-law or Mori–Tanaka scheme, which are two popular material models used in the literature. The FGM plates under consideration are made of aluminum (Al) and ceramic (zirconia (ZrO_2_) or alumina (Al_2_O_3_)), the material properties of which are given in Table 1.

**Figure 2:**
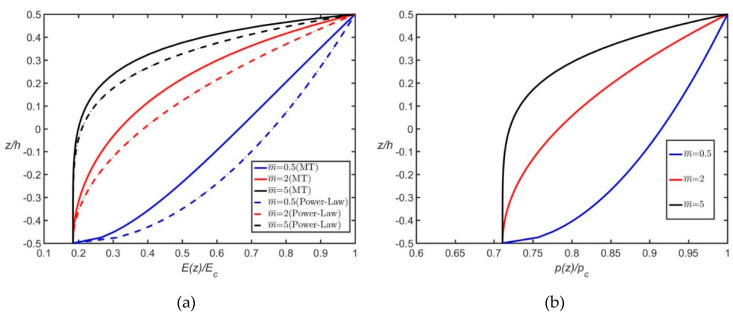
Variations of *E*(z) and ρ(z) for Al/Al_2_O_3_ through the thickness: (**a**) *E*(z); (**b**) ρ(z).

A power-law distribution of material properties is often assumed for FGMs. The material properties (i.e., Young’s modulus (E=E(z)), Poisson’s ratio (ν(z)), and mass density (ρ=ρ(z))) along the thickness of an FGM plate are given as follows:

(1) $$P(z)=P_{b}+V(z)(P_{t}-P_{b})=P_{b}+V(z)\Delta P$$

where $V(z)={\left(\frac{z}{h}+\frac{1}{2}\right)}^{\bar{m}}$; *P_b_* and *P_t_* denote the material properties at the bottom face (*z* = −*h*/2) and top face (*z* = *h*/2), respectively; ΔP is the difference between *P_b_* and *P_t_*, and $\bar{m}$ is the material property gradient index that governs the material variation profile in the thickness direction. Equation (1) indicates that if *P_b_* = *P_t_* or $\bar{m}$ = 0, *P*(*z*) is constant. FGM plates consisting of Al and ZrO_2_ (or Al_2_O_3_) exhibit a constant Poisson’s ratio because Al and ZrO_2_ (or Al_2_O_3_) have the same Poisson’s ratio. Figure 2 illustrates the distributions of E(z) and ρ(z) along the thickness of the Al/Al_2_O_3_ plates when $\bar{m}$ = 0.5, 2, and 5, where *E_c_* and $\rho_{c}$ are the Young’s modulus and density of Al_2_O_3_, respectively.

The Mori–Tanaka scheme is also frequently used to describe the material properties of FGMs. The effective mass density along the thickness of an FGM plate is given by

(2) $$\rho (z)=\rho_{1}V_{1}(z)+\rho_{2}V_{2}(z)$$

(3) $$V_{1}(z)+V_{2}(z)=1$$

(4) $$V_{1}(z)=V_{1}^{b}+(V_{1}^{t}-V_{1}^{b})(\frac{z}{h}+\frac{1}{2}{)}^{\bar{m}}$$

where subscripts 1 and 2 indicate materials 1 and 2, respectively, and $V_{1}^{t}$ and $V_{1}^{b}$ are the volume fractions of material 1 on the top and bottom surfaces of the plate, respectively. The effective local bulk modulus *K* and the shear modulus *G* are given by

(5) $$\frac{K(z)-K_{1}}{K_{2}-K_{1}}=\frac{V_{2}(z)}{1+\frac{\left(K_{2}-K_{1})V_{1}(z\right)}{K_{1}+(4/3)G_{1}}},\frac{G(z)-G_{1}}{G_{2}-G_{1}}=\frac{V_{2}(z)}{1+\frac{(G_{2}-G_{1})V_{1}(z)}{G_{1}+f_{1}}}$$

where $f_{1}=\frac{G_{1}(9K_{1}+8G_{1})}{6(K_{1}+2G_{1})}$. After the effective moduli *K* and *G* are estimated, the effective Young’s modulus and Poisson’s ratio are obtained using the following equation:

(6) $$E(z)=\frac{9K(z)G(z)}{3K(z)+G(z)}\;\mathrm{and}\;\nu (z)=\frac{3K(z)-2G(z)}{2(3K(z)+G(z))}$$

In the Mori–Tanaka scheme, the Poisson’s ratio is a function of *z* even when materials 1 and 2 have the same Poisson’s ratio. Equation (2) can be converted to the form of Equation (1), so the density distribution based on the Mori–Tanaka scheme is the same as that described by Equation (1). The distributions of *E*(z) and ρ(z) along the thickness of Al/Al_2_O_3_ plates with $\bar{m}$ = 0.5, 2, and 5 are illustrated in Figure 2 and denoted by “M–T”.

### 2.2. Governing Equations and Boundary Conditions

In the Mindlin plate theory [32], the displacement components of a plate are expressed as follows:

(7) $$\begin{array}{c} \bar{u}(x,y,z,t)=u_{0}(x,y,t)+z\psi_{x}(x,y,t),\;\bar{v}(x,y,z,t)=v_{0}(x,y,t)+z\psi_{y}(x,y,t),\\ \bar{w}(x,y,z,t)=w_{0}(x,y,t) \end{array}$$

where $\bar{u},\;\bar{v},\;\mathrm{and}\;\bar{w}$ are the displacement components in the *x*-, *y*-, and *z*-directions, respectively; $u_{0}$, $v_{0}$, and $w_{0}$ are the displacements on the mid-plane, and $\psi_{x}$ and $\psi_{y}$ are the rotations of the mid-plane normal in the *x*- and *y*-directions, respectively. The stress resultants are defined as follows:

(8) $$Q_{\beta }=\int_{-h/2}^{h/2}\sigma_{\beta z}dz,\;\left\{\begin{array}{c} N_{\beta \beta }\\ M_{\beta \beta } \end{array}\right\}=\int_{-h/2}^{h/2}\sigma_{\beta \beta }\left\{\begin{array}{c} 1\\ z \end{array}\right\}dz,\;\left\{\begin{array}{c} N_{xy}\\ M_{xy} \end{array}\right\}=\int_{-h/2}^{h/2}\sigma_{xy}\left\{\begin{array}{c} 1\\ z \end{array}\right\}dz$$

where the subscript  β represents x or y, and $\sigma_{ij}$ represents the stress components. The equations of motion are given as follows:

(9) $$\begin{array}{c} N_{xx,x}+N_{xy,y}=I_{0}{\ddot{u}}_{0}+I_{1}{\ddot{\psi }}_{x},\;N_{xy,x}+N_{yy,y}=I_{0}{\ddot{v}}_{0}+I_{1}{\ddot{\psi }}_{y},Q_{x,x}+Q_{y,y}=I_{0}{\ddot{w}}_{0},\\ M_{xx,x}+M_{xy,y}-Q_{x}=I_{1}{\ddot{u}}_{0}+I_{2}{\ddot{\psi }}_{x},\;M_{xy,x}+M_{yy,y}-Q_{y}=I_{1}{\ddot{v}}_{0}+I_{2}{\ddot{\psi }}_{y} \end{array}$$

where $I_{l}=\int_{-h/2}^{h/2}\rho (z)z^{l}dz\;(l=0,\;1\;\mathrm{and}\;2)$. The subscript comma denotes the partial derivative with respect to the coordinates defined by the variable after the comma.

Substituting linear strain–displacement (Equation (10)) and stress–strain relationships (Equation (11)) into Equation (8) yields the expressions for stress resultants in terms of displacement related components (Equation (12)).

(10) $$\varepsilon_{xx}=\frac{\partial \bar{u}}{\partial x},\;\varepsilon_{yy}=\frac{\partial \bar{v}}{\partial y},\;\varepsilon_{xy}=\frac{1}{2}(\frac{\partial \bar{v}}{\partial x}+\frac{\partial \bar{u}}{\partial y}),\;\varepsilon_{yz}=\frac{1}{2}(\frac{\partial \bar{w}}{\partial y}+\frac{\partial \bar{v}}{\partial z}),\;\varepsilon_{zx}=\frac{1}{2}(\frac{\partial \bar{u}}{\partial z}+\frac{\partial \bar{w}}{\partial x})$$

(11) $$\left\{\begin{array}{c} \begin{array}{c} \sigma_{xx}\\ \sigma_{yy} \end{array}\\ \sigma_{xy}\\ \sigma_{yz}\\ \sigma_{zx} \end{array}\right\}=\left[\begin{array}{ccccc} \frac{E}{1-\nu^{2}} & \frac{\nu E}{1-\nu^{2}} & 0 & 0 & 0\\ \frac{\nu E}{1-\nu^{2}} & \frac{E}{1-\nu^{2}} & 0 & 0 & 0\\ 0 & 0 & 2G & 0 & 0\\ 0 & 0 & 0 & 2G & 0\\ 0 & 0 & 0 & 0 & 2G \end{array}\right]\left\{\begin{array}{c} \varepsilon_{xx}\\ \varepsilon_{yy}\\ \varepsilon_{xy}\\ \varepsilon_{yz}\\ \varepsilon_{zx} \end{array}\right\}$$

(12) $$\begin{array}{c} N_{xx}={\bar{E}}_{0}u_{0,x}+{\bar{E}}_{1}\psi_{x,x}+{\bar{D}}_{0}v_{0,y}+{\bar{D}}_{1}\psi_{y,y},N_{yy}={\bar{D}}_{0}u_{0,x}+{\bar{D}}_{1}\psi_{x,x}+{\bar{E}}_{0}v_{0,y}+{\bar{E}}_{1}\psi_{y,y},\\ N_{xy}={\bar{G}}_{0}(u_{0,y}+v_{0,x})+{\bar{G}}_{1}(\psi_{x,y}+\psi_{y,x}),M_{xx}={\bar{E}}_{1}u_{0,x}+{\bar{E}}_{2}\psi_{x,x}+{\bar{D}}_{1}v_{0,y}+{\bar{D}}_{2}\psi_{y,y},\\ M_{yy}={\bar{D}}_{1}u_{0,x}+{\bar{D}}_{2}\psi_{x,x}+{\bar{E}}_{1}v_{0,y}+{\bar{E}}_{2}\psi_{y,y},\;M_{xy}={\bar{G}}_{1}(u_{0,y}+v_{0,x})+{\bar{G}}_{2}(\psi_{x,y}+\psi_{y,x}),\\ Q_{x}=\kappa {\bar{G}}_{0}(w_{0}{}_{,x}+\psi_{x}),\;Q_{y}=\kappa {\bar{G}}_{0}(w_{0}{}_{,y}+\psi_{y}) \end{array}$$

where

$${\bar{G}}_{i}=\int_{-h/2}^{h/2}Gz^{i}dz,\;{\bar{E}}_{i}=\int_{-h/2}^{h/2}\frac{E}{1-\nu^{2}}z^{i}dz\;\mathrm{and}\;{\bar{D}}_{i}=\int_{-h/2}^{h/2}\frac{\nu E}{1-\nu^{2}}z^{i}dz$$

The parameter κ is the transverse shear correction coefficient and is taken as 5/6 in the following analyses.

By substituting Equation (12) into Equation (9), the governing equations are obtained in terms of the displacement functions as follows:

(13) $${\bar{E}}_{0}u_{0,xx}+{\bar{E}}_{1}\psi_{x,xx}+{\bar{D}}_{0}v_{0,xy}+{\bar{D}}_{1}\psi_{y,xy}+{\bar{G}}_{0}(u_{0,yy}+v_{0,xy})+{\bar{G}}_{1}(\psi_{x,yy}+\psi_{y,xy})=I_{0}{\ddot{u}}_{0}+I_{1}{\ddot{\psi }}_{x}$$

(14) $${\bar{D}}_{0}u_{0,xy}+{\bar{D}}_{1}\psi_{x,xy}+{\bar{E}}_{0}v_{0,yy}+{\bar{E}}_{1}\psi_{y,yy}+{\bar{G}}_{0}(u_{0,xy}+v_{0,xx})+{\bar{G}}_{1}(\psi_{x,xy}+\psi_{y,xx})=I_{0}{\ddot{v}}_{0}+I_{1}{\ddot{\psi }}_{y}$$

(15) $$\kappa {\bar{G}}_{0}\left[\left(w_{0,xx}+\psi_{x,x})+(w_{0,yy}+\psi_{y,y}\right)\right]=I_{0}{\ddot{w}}_{0}$$

(16) $$\begin{array}{c} {\bar{E}}_{1}u_{0,xx}+{\bar{E}}_{2}\psi_{x,xx}+{\bar{D}}_{1}v_{0,xy}+{\bar{D}}_{2}\psi_{y,xy}+{\bar{G}}_{1}(u_{0,yy}+v_{0,xy})\\ +{\bar{G}}_{2}(\psi_{x,yy}+\psi_{y,xy})-\kappa {\bar{G}}_{0}(w_{0,x}+\psi_{x})=I_{1}{\ddot{u}}_{0}+I_{2}{\ddot{\psi }}_{x} \end{array}$$

(17) $$\begin{array}{c} {\bar{D}}_{1}u_{0,xy}+{\bar{D}}_{2}\psi_{x,xy}+{\bar{E}}_{1}v_{0,yy}+{\bar{E}}_{2}\psi_{y,yy}+{\bar{G}}_{1}(u_{0,xy}+v_{0,xx})\\ +{\bar{G}}_{2}(\psi_{x,xy}+\psi_{y,xx})-\kappa {\bar{G}}_{0}(w_{0,y}+\psi_{y})=I_{1}{\ddot{v}}_{0}+I_{2}{\ddot{\psi }}_{y} \end{array}$$

Each edge of a rectangular plate is simply supported (S), clamped (C) or free (F). For the edge with *y* = constant, the S, C and F boundary conditions are defined as follows:

Simply supported: $u_{0}=w_{0}=\psi_{x}=N_{yy}=M_{yy}=0$;
Clamped: $u_{0}$ = $v_{0}$ = $w_{0}$ = $\psi_{x}$ = $\psi_{y}$ = 0, and
Free: $N_{yy}=N_{xy}=Q_{y}=M_{yy}=M_{xy}=0$.

Similar definitions of boundary conditions are also applicable for the edge with *x* = constant.

### 2.3. Series Solutions

To establish the Fourier cosine series solutions for the vibrations of plates, let

(18) $$\begin{array}{c} u_{0}(x,y,t)=U_{0}(x,y)\cdot e^{i\omega t},\;v_{0}(x,y,t)=V_{0}(x,y)\cdot e^{i\omega t},\;w_{0}(x,y,t)=W_{0}(x,y)\cdot e^{i\omega t},\\ \psi_{x}(x,y,t)=\Psi_{x}(x,y)\cdot e^{i\omega t},\;\psi_{y}(x,y,t)=\Psi_{y}(x,y)\cdot e^{i\omega t} \end{array}$$

And

(19) $$U_{0}(x,y)=\sum_{m=0}^{M}\sum_{n=0}^{N}A_{mn}^{\left(1\right)}\;\cos\alpha_{m}x\;\cos\beta_{n}y+\sum_{l=1}^{2}\xi_{l}^{}(x)\sum_{n=0}^{N}B_{ln}^{\left(1\right)}\cos\beta_{n}y+\sum_{l=1}^{2}\eta_{l}^{}(y)\sum_{m=0}^{M}C_{lm}^{\left(1\right)}\cos\alpha_{m}x$$

(20) $$V_{0}(x,y)=\sum_{m=0}^{M}\sum_{n=0}^{N}A_{mn}^{\left(2\right)}\;\cos\alpha_{m}x\;\cos\beta_{n}y+\sum_{l=1}^{2}\xi_{l}^{}(x)\sum_{n=0}^{N}B_{ln}^{\left(2\right)}\cos\beta_{n}y+\sum_{l=1}^{2}\eta_{l}^{}(y)\sum_{m=0}^{M}C_{lm}^{\left(2\right)}\cos\alpha_{m}x$$

(21) $$W_{0}(x,y)=\sum_{m=0}^{M}\sum_{n=0}^{N}A_{mn}^{\left(3\right)}\;\cos\alpha_{m}x\;\cos\beta_{n}y+\sum_{l=1}^{2}\xi_{l}^{}(x)\sum_{n=0}^{N}B_{ln}^{\left(3\right)}\cos\beta_{n}y+\sum_{l=1}^{2}\eta_{l}^{}(y)\sum_{m=0}^{M}C_{lm}^{\left(3\right)}\cos\alpha_{m}x$$

(22) $$\Psi_{x}(x,y)=\sum_{m=0}^{M}\sum_{n=0}^{N}A_{mn}^{\left(4\right)}\;\cos\alpha_{m}x\;\cos\beta_{n}y+\sum_{l=1}^{2}\xi_{l}^{}(x)\sum_{n=0}^{N}B_{ln}^{\left(4\right)}\cos\beta_{n}y+\sum_{l=1}^{2}\eta_{l}^{}(y)\sum_{m=0}^{M}C_{lm}^{\left(4\right)}\cos\alpha_{m}x$$

(23) $$\Psi_{y}(x,y)=\sum_{m=0}^{M}\sum_{n=0}^{N}A_{mn}^{\left(5\right)}\;\cos\alpha_{m}x\;\cos\beta_{n}y+\sum_{l=1}^{2}\xi_{l}^{}(x)\sum_{n=0}^{N}B_{ln}^{\left(5\right)}\cos\beta_{n}y+\sum_{l=1}^{2}\eta_{l}^{}(y)\sum_{m=0}^{M}C_{lm}^{\left(5\right)}\cos\alpha_{m}x$$

where $\alpha_{m}=m\pi /a$, $\beta_{n}=n\pi /b$, and $\xi_{l}^{}\left(x\right)$ and $\eta_{l}^{}\left(y\right)$ are supplementary functions.

Tolstov [31] showed the following theorem on the differentiation of the Fourier series of a function:

**Theorem** **1.** *Let f(x) be a continuous function and have an absolutely integrable derivative on [0, L]. When f(x) is expanded as*

(24) $$f(x)=\sum_{n=1}^{\infty }{\tilde{b}}_{n}\sin\lambda_{n}x,\;\mathrm{where}\;\lambda_{n}=n\pi /L,\;$$

(25) $$f^{\prime }(x)=\frac{f(L)-f(0)}{L}+\sum_{n=1}^{\infty }\{\frac{2}{L}[{\left(-1\right)}^{n}f(L)-f(0)]+\lambda_{n}{\tilde{b}}_{n}\}\cos\lambda_{n}x$$

*When f(x) is expanded as*

(26) $$f(x)={\tilde{a}}_{0}+\sum_{n=1}^{\infty }{\tilde{a}}_{n}\cos\lambda_{n}x,\;f^{\prime }(x)=-\sum_{n=1}^{\infty }\lambda_{n}{\tilde{a}}_{n}\sin\lambda_{n}x$$

The theorem indicates that the Fourier cosine series can be differentiated term-by-term, while such an operation can be applied to the Fourier sine series only if *f*(0) = *f*(*L*) = 0. To remedy such shortcoming of the sine series, Li [33] proposed to add some supplementary functions into the cosine series in Equation (26) and to determine $f^{\prime \prime }(x)$ and $f^{\left(iv\right)}(x)$ via term-by-term differential.

According to Li [33,34], the supplementary functions are typically determined by satisfying the following conditions:

(27) $$\begin{array}{c} \xi_{1,x}(0)=1,\;\xi_{1,x}(a)=0,\;\xi_{2,x}(0)=0\;,\;\xi_{2,x}(a)=1\;,\;\eta_{1,y}(0)=1,\;\eta_{1,y}(b)=0,\;\eta_{2,y}(0)=0\;,\\ \eta_{2,y}(b)=1,\;\int_{0}^{a}\xi_{l}(x)dx=0\;\mathrm{and}\;\int_{0}^{b}\eta_{l}^{}(y)dy=0\;(l\;=\;1,\;2). \end{array}$$

If polynomial functions are used, Equation (27) leads to

(28) $$\xi_{1}(x)=-\frac{x^{2}}{2a}+x-\frac{a}{3},\;\xi_{2}(x)=\frac{x^{2}}{2a}-\frac{a}{6},\;\eta_{1}(y)=-\frac{y^{2}}{2b}+y-\frac{b}{3},\;\eta_{2}(y)=\frac{y^{2}}{2b}-\frac{b}{6}.$$

Substituting Equations (18)–(23) and (28) into the boundary conditions yields a set of linear algebraic equations for the coefficients $A_{mn}^{\left(i\right)}$, $B_{ln}^{\left(i\right)}$ and $C_{ln}^{\left(i\right)}$. For instance, when $U_{0}(a,y)$ = 0, which is one of the fixed boundary conditions at *x* = *a*, the following equation is obtained:

(29) $$\sum_{n=0}^{N}[\sum_{m=0}^{M}A_{mn}^{\left(1\right)}\;\cos\alpha_{m}a\;+\sum_{l=1}^{2}\xi_{l}^{}(a)B_{ln}^{\left(1\right)}+\sum_{l=1}^{2}{\bar{c}}_{ln}\sum_{m=0}^{M}C_{lm}^{\left(1\right)}\cos\alpha_{m}a]\cos\beta_{n}y=0$$

In establishing Equation (29), $\eta_{l}\left(y\right)$ is expressed in its Fourier cosine series as follows:

(30) $$\eta_{l}(y)=\sum_{n=0}^{N}{\bar{c}}_{ln}\cos\beta_{n}y$$

where ${\bar{c}}_{ln}=\int_{0}^{b}\eta_{l}(y)\cos\beta_{n}ydy/\int_{0}^{b}(\cos\beta_{n}y{)}^{2}dy$. Equation (29) includes *N* + 1 functions of $\cos\beta_{n}y$, and each coefficient of $\cos\beta_{n}y$ must equal zero in order to satisfy the equation. Consequently, Equation (29) provides *N* + 1 linear algebraic equations for the coefficients $A_{mn}^{\left(1\right)}$, $B_{ln}^{\left(1\right)}$ and $C_{ln}^{\left(1\right)}$,

(31) $$\sum_{m=0}^{M}\;A_{mn}^{\left(1\right)}\cos\alpha_{m}a\;+\sum_{l=1}^{2}\xi_{l}^{}(a)B_{ln}^{\left(1\right)}+\sum_{l=1}^{2}{\bar{c}}_{\ln}\sum_{m=0}^{M}C_{lm}^{\left(1\right)}\cos\alpha_{m}a=0\;(n=0,\;1,\;2,\;\cdots ,\;N)$$

Similarly, one can establish 10 (*M* + *N* + 2) linear algebraic homogeneous equations for $A_{mn}^{\left(i\right)}$, $B_{ln}^{\left(i\right)}$ and $C_{ln}^{\left(i\right)}$ from the boundary conditions along the four edges of a rectangular plate. Such set of equations can be further expressed in the matrix form as follows:

(32) $$B_{p}\;P=B_{A}\;A$$

where $P={\left(B_{ln}^{\left(1\right)}\;C_{lm}^{\left(1\right)}\;B_{ln}^{\left(2\right)}\;C_{lm}^{\left(2\right)}\cdots B_{ln}^{\left(5\right)}\;C_{lm}^{\left(5\right)}\right)}^{T}$ and $A={\left(A_{mn}^{\left(1\right)}\;A_{mn}^{\left(2\right)}\cdots A_{mn}^{\left(5\right)}\right)}^{T}$ (l=1,2; m=0,1,⋯M; n=0,1,⋯N).

To satisfy the governing equations, substituting Equations (18)–(23) and (28) into Equations (13)–(17) also yields a set of linear algebraic equations for the coefficients $A_{mn}^{\left(i\right)}$, $B_{ln}^{\left(i\right)}$ and $C_{ln}^{\left(i\right)}$. For example, Equation (13) yields the following equation:

(33) $$\begin{array}{l} \sum_{m=0}^{M}\sum_{n=0}^{N}\{(-\alpha_{m}^{2}{\bar{E}}_{0}-\beta_{n}^{2}{\bar{G}}_{0})A_{mn}^{\left(1\right)}+\sum_{l=1}^{2}(-\beta_{n}^{2}{\bar{G}}_{0}{\tilde{C}}_{lm}+{\bar{E}}_{0}{\tilde{C}}_{lm}^{\left(2\right)})B_{ln}^{\left(1\right)}+\sum_{l=1}^{2}(-\alpha_{m}^{2}{\bar{E}}_{0}{\tilde{J}}_{ln}+{\bar{G}}_{0}{\tilde{J}}_{ln}^{\left(2\right)})C_{lm}^{\left(1\right)}+\\ \;\sum_{i=0}^{M}\sum_{j=0}^{N}\alpha_{i}^{}\beta_{j}^{}({\bar{D}}_{0}+{\bar{G}}_{0}){\bar{S}}_{mn}^{\left(ij\right)}A_{ij}^{\left(2\right)}+\sum_{j=0}^{N}\sum_{l=1}^{2}-\beta_{j}^{}({\bar{D}}_{0}+{\bar{G}}_{0}){\tilde{C}}_{lm}^{\left(1\right)}{\tilde{S}}_{yn}^{\left(j\right)}B_{lj}^{\left(2\right)}+\\ \;\sum_{i=0}^{M}\sum_{l=1}^{2}-\alpha_{i}^{}({\bar{D}}_{0}+{\bar{G}}_{0}){\tilde{J}}_{ln}^{\left(1\right)}{\tilde{S}}_{xn}^{\left(i\right)}C_{li}^{\left(2\right)}+(-\alpha_{m}^{2}{\bar{E}}_{1}-\beta_{n}^{2}{\bar{G}}_{1})A_{mn}^{\left(4\right)}+\sum_{l=1}^{2}({\bar{E}}_{1}{\tilde{C}}_{lm}^{\left(2\right)}-\beta_{n}^{2}{\bar{G}}_{1}{\tilde{C}}_{lm})B_{ln}^{\left(4\right)}+\\ \;\sum_{l=1}^{2}(-\alpha_{m}^{2}{\bar{E}}_{1}{\tilde{J}}_{ln}+{\bar{G}}_{1}{\tilde{J}}_{ln}^{\left(2\right)})C_{lm}^{\left(4\right)}+\sum_{i=0}^{M}\sum_{j=0}^{N}\alpha_{i}^{}\beta_{j}^{}({\bar{D}}_{1}+{\bar{G}}_{1}){\bar{S}}_{mn}^{\left(ij\right)}A_{ij}^{\left(5\right)}+\\ \;\sum_{j=0}^{N}\sum_{l=1}^{2}-\beta_{j}^{}({\bar{D}}_{1}+{\bar{G}}_{1}){\tilde{C}}_{lm}^{\left(1\right)}{\tilde{S}}_{yn}^{\left(j\right)}B_{lj}^{\left(5\right)}+\sum_{i=0}^{M}\sum_{l=1}^{2}-\alpha_{i}^{}({\bar{D}}_{1}+{\bar{G}}_{1}){\tilde{J}}_{ln}^{\left(1\right)}{\tilde{S}}_{xn}^{\left(i\right)}C_{li}^{\left(5\right)}\}\cos\alpha_{m}x\;\cos\beta_{n}y=\\ -\omega^{2}\sum_{m=0}^{M}\sum_{n=0}^{N}\{\;I_{0}A_{mn}^{\left(1\right)}+I_{1}A_{mn}^{\left(4\right)}+\sum_{l=1}^{2}{\tilde{C}}_{lm}(I_{0}B_{ln}^{\left(1\right)}+I_{1}B_{ln}^{\left(4\right)})+\sum_{l=1}^{2}{\tilde{J}}_{ln}(I_{0}C_{lm}^{\left(1\right)}+I_{1}C_{lm}^{\left(4\right)})\}\cos\alpha_{m}x\;\cos\beta_{n}y. \end{array}$$

In establishing Equation (33), the following functions are expressed in terms of the Fourier cosine series to factor out $\cos\alpha_{m}x\;\cos\beta_{n}y$:

(34) $$\begin{array}{c} \xi_{l}(x)=\sum_{m=0}^{M}{\tilde{C}}_{lm}\cos\alpha_{m}x,\;\xi_{l,x}(x)=\sum_{m=0}^{M}{\tilde{C}}_{lm}^{\left(1\right)}\cos\alpha_{m}x,\;\xi_{l,xx}(x)=\sum_{m=0}^{M}{\tilde{C}}_{lm}^{\left(2\right)}\cos\alpha_{m}x\\ \eta_{l}(y)=\sum_{n=0}^{N}{\tilde{J}}_{ln}\cos\beta_{n}y,\;,\;\eta_{l,y}(y)=\sum_{n=0}^{N}{\tilde{J}}_{ln}^{\left(1\right)}\cos\beta_{n}y,\;\eta_{l,yy}(y)=\sum_{n=0}^{N}{\tilde{J}}_{ln}^{\left(2\right)}\cos\beta_{n}y,\;\\ \sin\alpha_{i}x=\sum_{m=0}^{M}{\tilde{S}}_{xm}^{\left(i\right)}\cos\alpha_{m}x,\;\sin\beta_{i}y=\sum_{n=0}^{N}{\tilde{S}}_{yn}^{\left(i\right)}\cos\beta_{n}y,\;\\ \sin\alpha_{i}x\;\sin\beta_{j}y=\sum_{m=0}^{M}\sum_{n=0}^{N}{\tilde{S}}_{mn}^{\left(ij\right)}\cos\alpha_{m}x\;\cos\beta_{n}y. \end{array}$$

Similarly, one can establish 5 (*M* + 1) (*N* + 1) linear algebraic homogeneous equations for $A_{mn}^{\left(i\right)}$, $B_{ln}^{\left(i\right)}$ and $C_{ln}^{\left(i\right)}$ from the governing equations (Equations (13)–(17)). Such set of equations can be further expressed in the following matrix form:

(35) $$\left(\hat{K}A+\tilde{K}P\right)-\omega^{2}\left(\hat{M}A+\tilde{M}P\right)=0$$

Equation (32) gives

(36) $$P=(B_{p}^{-1}\;B_{A})\;A=\Gamma A$$

Substituting Equation (36) into Equation (35) yields

(37) $$\left(\hat{K}+\tilde{K}\Gamma \right)A=\omega^{2}\left(\hat{M}+\tilde{M}\Gamma \right)A$$

which forms an eigenvalue problem.

## 3. Convergence Studies and Comparisons

The boundary conditions for a rectangular plate at the edges *x* = 0, *y* = 0, *x* = *a* and *y* = b are specified by four letters in a respective series. For example, CSFF boundary conditions mean a clamped boundary condition at *x* = 0, a simply supported boundary condition at *y* = 0 and a free boundary condition at *x* = *a* and *y* = b. To validate the proposed solutions, comprehensive convergence studies were carried out for the nondimensional vibration frequencies $\Omega \;(=\omega (a^{2}/h)\sqrt{\rho_{c}/E_{c}},$ where the subscript “*c*” indicates ceramic material properties) of the first six modes of square plates with *h*/*b* = 0.1 and with SSSS, SCSC, CFFF and FFFF boundary conditions. The obtained results were compared with the published results from the literature. In the following, the solution terms *M* and *N* in Equations (19)–(23) are set equal, and $\bar{M}\;(=M+1)$ mainly equals 5, 10, 15, 25, 30 and 35 in the convergence studies. An Al/Al_2_O_3_ FGM, whose material properties are described by Equation (1) (the power-law model), is mainly considered.

Table 2 presents the convergence studies for Al/Al_2_O_3_ and Al/ZrO_2_ FGM ($\bar{m}=1$) plates with SSSS boundary conditions. Notably, the material properties of the Al/ZrO_2_ FGM are determined by the Mori–Tanaka scheme. In the following tables, the mode denoted by “*” is the in-plane displacement-dominated mode. The results determined from simple exact closed-form solutions (see Appendix A) are given, and the superscripts “(*m*, *n*)” denote the wave numbers in the *x*-direction and *y*-direction, respectively. Some published results based on the Mindlin plate theory are also given in Table 2 for comparison. The published results presented in the table include (1) the results provided by Hosseini-Hashemi et al. [23], who proposed exact analytical solutions for FGM rectangular plates having two simply supported opposite edges; (2) the numerical results of Zhao et al. [10], who obtained solutions by using an element-free *kp*-Ritz method with shape functions constructed based on the kernel particle concept; (3) the numerical results of Ferreira et al. [12], who used the global collocation method with multi-quadric radial basis functions.

**Figure 3:**
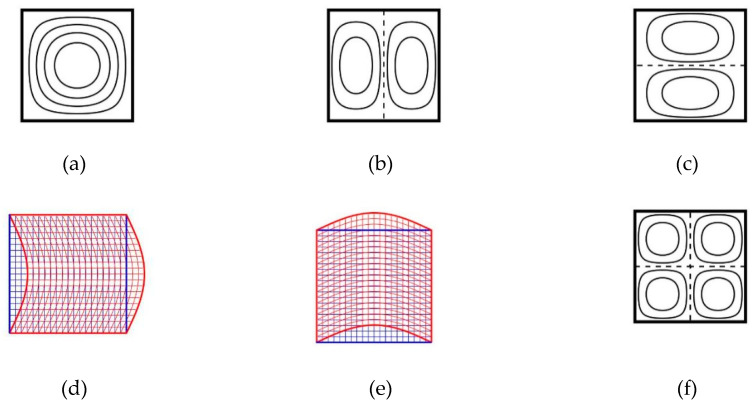
Mode shapes for an FGM square plate with SSSS boundary conditions: (**a**) mode 1; (**b**) mode 2; (**c**) mode 3; (**d**) mode 4; (**e**) mode 5; (**f**) mode 6.

The present results converge from the upper-bounds of solutions as the number of solution terms increases. The results obtained using $\bar{M}$ = 15 show the agreement of three significant figures with the results determined from the simple exact closed-form solutions given in Appendix A, and the differences are less than 0.1%. It is interesting to observe that although the solutions of Hosseini-Hashemi et al. [23] provided accurate results for out-of-plane displacement-dominated modes, they did not provide the results for a wave number of zero in the *x*-direction or *y*-direction, which corresponded to the in-plane displacement-dominated modes. The first six modal shapes are depicted in Figure 3. In this figure, the contours of out-of-plane displacement ($W_{0}$) (represented by solid lines) and the nodal lines (represented by dashed lines) are depicted for the out-of-plane flexural modes. Moreover, the in-plane modal deformations are displayed for the in-plane displacement-dominated modes. Compared with the results obtained from the exact closed-form solutions, the results of Zhao et al. [10] exhibited a difference of approximately 1.6% and the results of Ferreira et al. [12] exhibited a difference between 0.9% and 3.3%. Consequently, the present results obtained using $\bar{M}$ = 15 are more accurate than those of Zhao et al. [10] and Ferreira et al. [12].

Table 3 lists the non-dimensional natural frequencies Ω obtained using $\bar{M}$ equal to 5, 10, 15, 25, 30 and 35 for FGM ($\bar{m}=$ 0 and 1) square plates with SCSC boundary conditions. As $\bar{M}$ increases, the natural frequencies converge from the upper-bounds of solutions. The results obtained using $\bar{M}$ = 15 and 35 show the consistency of three significant figures. The present results for the homogeneous plate ($\bar{m}=$ 0) obtained using $\bar{M}$ = 35 show excellent agreement of four significant figures with those of Liew et al. [35] and Du et al. [36], who investigated the vibrations of homogeneous rectangular plates. Liew et al. [34] determined the frequencies of out-of-plane modes via the conventional Ritz method with polynomial admissible functions, while Du et al. [36] established series solutions for the in-plane vibrations of rectangular plates. The present results for out-of-plane displacement-dominated modes obtained using $\bar{M}\geq$ 25 for the FGM ($\bar{m}=$ 0 and 1) square plates are consistent with the results of Hosseini-Hashemi et al. [23] for at least three significant figures. Again, the solutions of Hosseini-Hashemi et al. [23] neglected the in-plane mode for a wave number of zero in the *x*-direction.

Table 4 presents the convergence of the non-dimensional natural frequencies Ω of cantilevered FGM ($\bar{m}=$ 0 and 5) square plates with various numbers of solution terms. Interestingly, the results indicate different convergence trends from those observed from Table 1 and Table 2. The values of Ω for the homogeneous plate ($\bar{m}=$ 0) reveal convergence from the upper-bounds of solutions for the first and third modes, convergence from the lower-bounds of solutions for the second, fourth and sixth modes, and oscillatory convergence for the fifth mode. These findings are not applied to the results for the FGM plate with $\bar{m}=$ 5. For example, oscillatory convergence is observed for the first and fifth modes. The present results obtained using $\bar{M}$ ≥ 25 exhibit excellent agreement with those of Liew et al. [35] with the differences less than 0.1%. The differences between the present convergent results and those of Zhao et al. [10] can be larger than 1%.

Similar to Table 4, Table 5 considers the plates with FFFF boundary conditions. Notably, using supplementary functions given in Equation (28) yields singular **B**_p_ in Equation (32), and its inverse cannot be found for Equation (36). To overcome such numerical difficulties, in addition to the conditions presented in Equation (27), the following conditions are proposed to establish the polynomial supplementary functions:

(38) $$\xi_{i}(0)=\xi_{i}(a)=\eta_{i}(0)=\eta_{i}(b)=0\;(\mathrm{for}\;i=\;1\;\mathrm{and}\;2).$$

Satisfying Equations (27) and (38) yields

(39) $$\begin{array}{l} \xi_{1}(x)=-\frac{5x^{4}}{2a^{3}}+\frac{6x^{3}}{a^{2}}-\frac{9x^{2}}{2a}+x,\;\xi_{2}(x)=\frac{5x^{4}}{2a^{3}}-\frac{4x^{3}}{a^{2}}+\frac{3x^{2}}{2a},\\ \eta_{1}(y)=-\frac{5y^{4}}{2b^{3}}+\frac{6y^{3}}{b^{2}}-\frac{9y^{2}}{2b}+y,\;\eta_{2}(y)=\frac{5y^{4}}{2b^{3}}-\frac{4y^{3}}{b^{2}}+\frac{3y^{2}}{2b} \end{array}$$

Table 5 lists the results obtained using $\bar{M}$ = 5, 15, 25, 35, 40 and 45. Notably, six rigid body modes with zero frequencies are not considered in the table. The convergence of the numerical results is slower than that of the results presented in Table 2, Table 3 and Table 4. When the results of the homogenous plate ($\bar{m}=$ 0) are under consideration, oscillatory convergence is found for the third to sixth modes, while convergence from lower-bounds and upper-bounds is observed for the first and second modes, respectively. The results obtained using $\bar{m}\geq$ 35 are consistent with those of Liew et al. [35] with the differences less than 0.1%.

The convergence behaviors of the results for the FGM plate with $\bar{m}=$5 and FFFF boundary conditions are different from those for the homogeneous plate. The convergence of natural frequencies for different modes is monotonic from the upper- or lower-bounds. The results obtained using $\bar{M}\geq$ 35 are in good agreement with those obtained from the Ritz method based on three-dimensional elasticity [20] with the differences less than 0.6%.

To simply demonstrate the convergence rates of the present solutions for other combinations of boundary conditions, Table 6 shows the average relative differences in the Ω values of the first six modes obtained using $\bar{M}=\bar{N}$ = 15 and $\bar{M}=\bar{N}$ = 35 for Al/Al_2_O_3_ FGM square plates with *h*/*a* = 0.1 and $\bar{m}=$ 5 at 16 combinations of boundary conditions. All the differences are less than 0.1%, which indicates that the solutions derived in this study provide accurate results even when $\bar{M}=\bar{N}$ = 15 is used. Differences larger than 0.08% occur in the results for SFSF and SFFF boundary conditions, while the differences are less than 0.03% when CSSF, CSCF, CCSF and CCCF boundary conditions are under consideration.

## 4. Numerical Results

**Figure 4:**
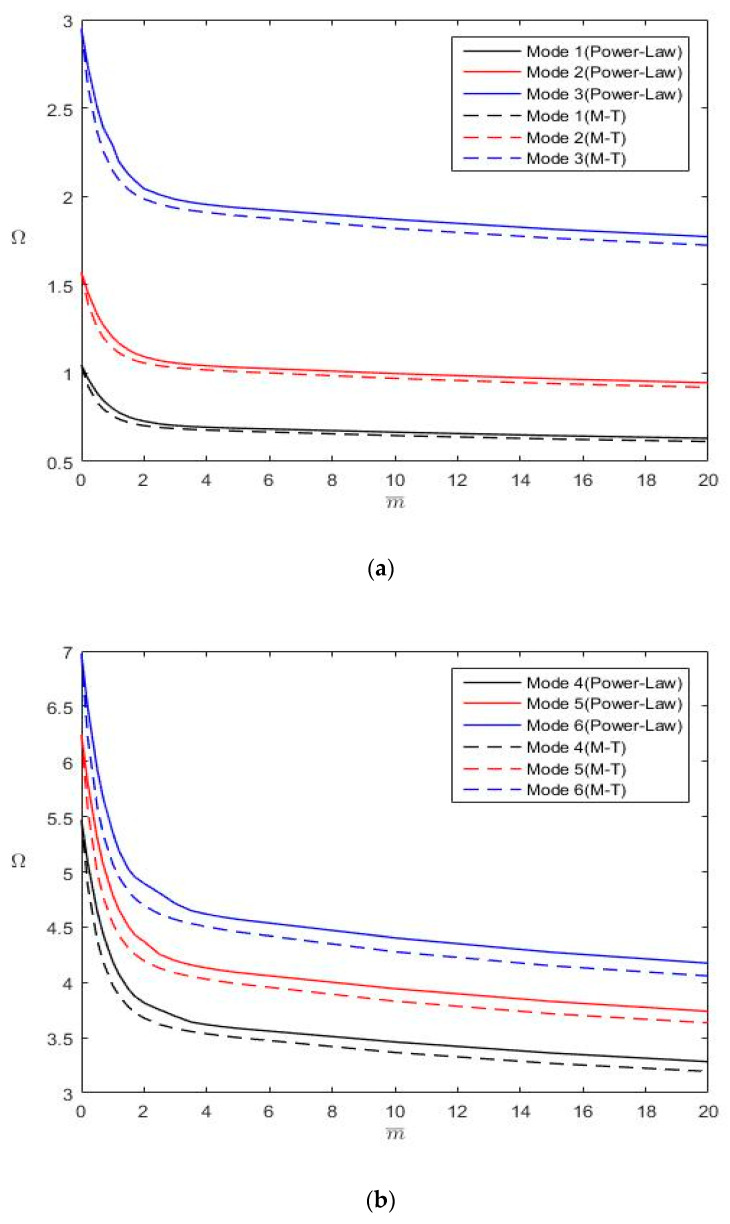
Variations of Ω with $\bar{m}$ for a CFFF FGM rectangular plate *(b*/*a* = 2, *h*/*a* = 0.1): (**a**) for modes 1–3; (**b**) for modes 4–6.

**Figure 5:**
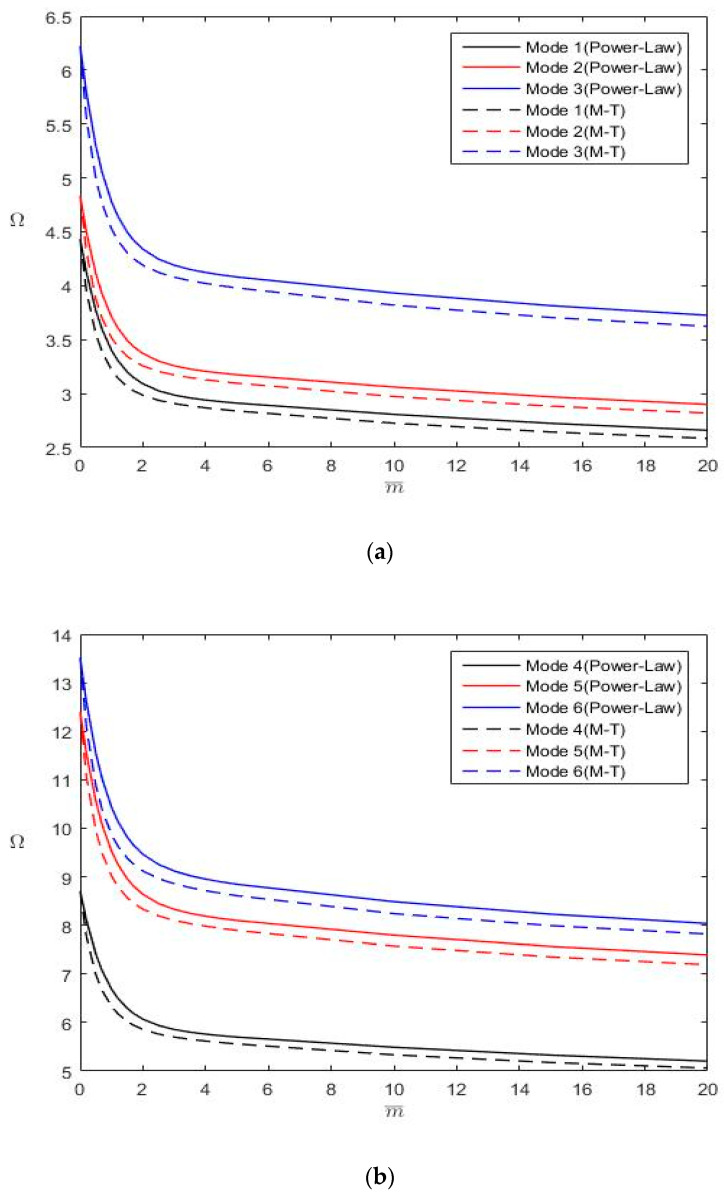
Variations of Ω with $\bar{m}$ for a CFSF FGM rectangular plate *(b*/*a* = 2, *h*/*a* = 0.1): (**a**) for modes 1–3; (**b**) for modes 4–6.

After validating the proposed analytical solutions through convergence studies, we employed the solutions to determine the first six nondimensional frequencies (Ω) of Al/Al_2_O FGM plates with various aspect ratios (*b*/*a* = 1 and 2), thickness-to-length ratios (*h*/*a* = 0.02 and 0.1), power-law index values ($\bar{m}=$ 0, 0.5, 2 and 5), and combinations of boundary conditions (CCCC, FFFF, CFFF, CFSF, SSFF, CSFF, CSSF, CCFF and CCCF). The results are summarized in Table 7, Table 8, Table 9 and Table 10, in which “*” denotes the in-plane displacement-dominated modes. Figure 4 and Figure 5 depict the variations in Ω with $\bar{m}$ for Al/Al_2_O FGM rectangular plates (*b*/*a* = 2 and *h*/*a* = 0.1) with CFFF and CFSF boundary conditions, respectively. These results were obtained using $\bar{M}=\bar{N}$ = 35, except for FFFF plates, whose natural frequencies were determined by using $\bar{M}=\bar{N}$ = 45. Since exact closed-form solutions exist for plates with two simply supported opposite edges, such boundary conditions are not under consideration in this section. These tabulated results can serve as benchmark data for evaluating numerical approaches.

The following inferences are drawn from Table 7, Table 8, Table 9 and Table 10 and Figure 4 and Figure 5:

1. The constraint increases when a free boundary condition changes to a simply supported boundary condition. The constraint further increases in a clamped boundary condition. Higher constraint results in higher plate stiffness and larger natural frequencies. Therefore, $\Omega_{\mathrm{CCCC}}$ > $\Omega_{\mathrm{CSSF}}$ > $\Omega_{\mathrm{CSFF}}$ > $\Omega_{\mathrm{SSFF}}$ and $\Omega_{\mathrm{CCCC}}$ > $\Omega_{\mathrm{CCCF}}$ > $\Omega_{\mathrm{CCFF}}$ > $\Omega_{\mathrm{CSFF}}$ > $\Omega_{\mathrm{CFFF}}$ > $\Omega_{\mathrm{FFFF}}$ (where the subscripts indicate the boundary conditions) if the first six rigid body modes with zero frequencies are considered for plates with FFFF boundary conditions.
2. The Mori–Tanaka material model provides a larger Young’s modulus than the power-law material model does; however, both models yield the same density distribution (Figure 2). Consequently, FGM plates following the Mori-Tanaka material model have larger natural frequencies than those following the power-law material model.
3. An increase in $\bar{m}$ results in a decrease in Ω. Furthermore, as displayed in Figure 4 and Figure 5, the change rate of Ω with $\bar{m}$ gradually decreases with an increase in $\bar{m}$. Notably, an increase in $\bar{m}$ leads to a decrease in the plate stiffness and mass (Figure 2).
4. No in-plane displacement-dominated mode exists in the first six modes for thin square plates with *h*/*a* = 0.02; however, such a mode may exist for moderately thick plates with *h*/*a* = 0.1.
5. The nondimensional frequencies (Ω) of plates with *h*/*a* = 0.1 are less than those of plates with *h*/*a* = 0.02 because *h*/*a* is involved in the definition of Ω. When converting Ω to ω, one finds that the trend is opposite for ω because the plate rigidity increases with *h*/*a*.

## 5. Concluding Remarks

In this study, analytical solutions based on the Mindlin plate theory were developed for the vibrations of FGM rectangular plates with various combinations of boundary conditions. The solutions were established using the Fourier cosine series with polynomial supplementary functions. Fourth-order polynomial supplementary functions were adopted in the solutions for FFFF boundary conditions, and second-order polynomial supplementary functions were adopted in the solutions for the other boundary conditions. The present solutions were validated through comprehensive convergence studies as well as comparisons with published results and the exact closed-form solutions for plates with SSSS boundary conditions. When increasing the number of solution terms, the trends of convergence in vibration frequencies (i.e., monotonous convergence from upper-bounds or lower-bounds or convergence in an oscillatory manner) varied according to the vibration modes, distributions of material properties, and boundary conditions. The vibration frequencies of the first six modes obtained using $\bar{M}=\bar{N}$ = 15 were in good agreement with those obtained using $\bar{M}=\bar{N}$ = 35, and the average differences were less than 0.1% for FGM square plates with *h*/*a* = 0.1 and $\bar{m}$ = 5 under 17 combinations of boundary conditions, excluding FFFF boundary conditions. The average difference was about 0.6% for FFFF boundary conditions.

The present solutions were also applied to determine the vibration frequencies of Al/Al_2_O FGM plates with CCCC, FFFF, CFFF, CFSF, SSFF, CSFF, CSSF, CCFF and CCCF boundary conditions. The effects of the plate thickness, material model (Mori–Tanaka and power-law models), and power-law index, $\bar{m}$ on the vibration frequencies were investigated. With a fixed $\bar{m}$, the Mori–Tanaka model yielded higher plate stiffness and larger vibration frequencies for plates than the power-law model. An increase in $\bar{m}$ caused a decrease in the vibration frequencies of Al/Al_2_O FGM plates. The tabulated data in this study can be used as a standard to judge the accuracy of numerical methods. The present solutions can be simply modified to determine the buckling loads of an FGM rectangular plate under uniform initial stresses and to perform linear static and dynamic analyses of an FGM rectangular plate.

## Figures and Tables

**Table 1:** Material properties of the FGM ingredients.

Material	Properties		
	*E* (GPa)	Poisson’s Ratio (*ν*)	ρ (kg/m^3^)
Aluminum (Al)	70.0	0.3	2702
Alumina (Al_2_O_3_)	380	0.3	3800
Zirconia (ZrO_2_)	200	0.3	5700

**Table 2:** Convergence of $\Omega =\omega (a^{2}/h)\sqrt{\rho_{c}/E_{c}}$ for SSSS FGM square plates with *h/b* = 0.1 and $\bar{m}=1$.

Material Model	Material Ingredient	Mode	$\bar{M}=M+1$						Exact Closed-Form Sol.	Published
			5	10	15	25	30	35		
Power-Law	Al/Al_2_O_3_	1	4.510	4.433	4.422	4.419	4.418	4.418	4.419 ^(1,1)^	<4.420> (4.347)
		2	11.03	10.63	10.60	10.60	10.58	10.58	10.59 ^(1,2)^	<10.59> (10.42)
		3	11.03	10.63	10.60	10.56	10.58	10.58	10.59 ^(2,1)^	</> (10.42)
		4 *	16.22	16.20	16.20	16.20	16.20	16.20	16.20 ^(1,0)^	<×> (15.94)
		5 *	16.22	16.20	16.20	16.20	16.20	16.20	16.20 ^(0,1^)	<×> (/)
		6	16.90	16.34	16.31	16.30	16.30	16.30	16.31 ^(2,2)^	<16.31> (/)
M-T	Al/ZrO_2_	1	5.288	5.205	5.193	5.190	5.190	5.190	5.192 ^(1,1)^	{5.096}
		2	12.90	12.45	12.42	12.41	12.41	12.41	12.41 ^(1,2)^	{12.30}
		3	12.90	12.45	12.42	12.41	12.41	12.41	12.41 ^(2,1)^	{12.30}
		4 *	18.10	18.09	18.08	18.08	18.08	18.08	18.08 ^(1,0)^	{17.49}
		5 *	18.10	18.09	18.08	18.08	18.08	18.08	18.08 ^(0,1)^	{17.49}
		6	19.74	19.12	19.08	19.07	19.06	19.06	19.09 ^(2,2)^	{18.87}

Note: ‘×’denotes data missed; ‘/’ denotes data not available; < > denotes results of Hosseini-Hashemi et al. [23]; ( ) denotes results of Zhao et al. [10]; { } denotes results of Ferreira et al. [12]; “*” denotes the in-plane displacement-dominated model; the superscripts “( )” denote the wave numbers in the x-direction and y-direction.

**Table 3:** Convergence of Ω for SCSC FGM square plates with *h/b* = 0.1.

$\bar{m}$	Mode	$\bar{M}=M+1$						Published
		5	10	15	25	30	35	
0	1	8.183	8.079	8.073	8.071	8.071	8.070	{8.070} <8.070>
	2	15.37	14.91	14.88	14.87	14.86	14.86	{14.86} <14.86>
	3	18.25	17.95	17.93	17.92	17.92	17.92	{17.92} <17.92>
	4 *	19.50	19.49	19.48	19.48	19.48	19.48	[19.48] < × >
	5	24.49	23.91	23.87	23.85	23.85	23.85	{23.85} <23.85>
	6	27.72	26.44	26.32	26.29	26.29	26.28	{26.28} </>
1	1	6.320	6.228	6.223	6.221	6.221	6.221	<6.220>
	2	11.89	11.51	11.48	11.47	11.47	11.47	<11.47>
	3	14.17	13.94	13.92	13.91	13.91	13.91	<13.92>
	4 *	16.22	16.20	16.20	16.20	16.20	16.20	<×>
	5	19.05	18.57	18.54	18.53	18.53	18.53	<18.54>
	6	21.62	20.48	20.38	20.35	20.35	20.35	</>

Note: ‘×’ denotes data missed; ‘/’ denotes data not available; { } denotes results of Liew et al. [35]; [] denotes results of Du et al. [36]; < > denotes results of Hosseini-Hashemi et al. [23]; “*” denotes the in-plane displacement-dominated mode.

**Table 4:** Convergence of Ω for CFFF FGM square plates with *h/b* = 0.1.

$\bar{m}$	Mode	$\bar{M}=M+1$						Published
		5	10	15	25	30	35	
0	1	1.039	1.038	1.038	1.038	1.038	1.038	{1.038} (1.030)
	2	2.399	2.428	2.435	2.438	2.439	2.439	{2.440} (2.391)
	3	6.134	6.082	6.079	6.079	6.079	6.079	{6.080} (6.005)
	4 *	6.548	6.576	6.578	6.580	6.581	6.581	{/} (7.636)
	5	7.742	7.702	7.712	7.715	7.716	7.716	{7.716} (/)
	6	8.417	8.518	8.533	8.544	8.545	8.546	{8.548} (/)
5	1	0.6833	0.6826	0.6827	0.6828	0.6828	0.6828	(0.6768)
	2	1.575	1.594	1.599	1.601	1.601	1.601	(1.568)
	3	4.017	3.983	3.981	3.981	3.981	3.981	(3.927)
	4 *	4.253	4.272	4.273	4.274	4.274	4.275	(4.263)
	5	5.065	5.039	5.045	5.047	5.047	5.047	(/)
	6	5.510	5.577	5.586	5.593	5.594	5.594	(/)

Note: ‘/’ denotes data not available; { } denotes results of Liew et al. [35]; ( ) denotes results of Zhao et al. [10]; “*” denotes the in-plane displacement-dominated mode.

**Table 5:** Convergence of Ω for FFFF FGM square plates with *h/b* = 0.1

$\bar{m}$	Mode	$\bar{M}=\bar{N}$						Published
		5	15	25	35	40	45	
0	1	3.823	3.842	3.846	3.847	3.849	3.849	{3.849}
	2	6.921	5.794	5.745	5.737	5.736	5.736	{5.733}
	3	7.821	7.091	7.064	7.059	7.060	7.060	{7.058}
	4	10.08	9.665	9.656	9.655	9.660	9.660	{9.660}
	5	10.08	9.665	9.656	9.655	9.660	9.660	{9.660}
	6	16.93	16.76	16.74	16.74	16.75	16.75	{16.75}
5	1	2.508	2.521	2.523	2.524	2.524	2.524	(2.512)
	2	4.516	3.790	3.759	3.753	3.752	3.752	(3.746)
	3	5.111	4.640	4.623	4.620	4.620	4.619	(4.608)
	4	6.579	6.314	6.309	6.308	6.308	6.308	(6.270)
	5	6.579	6.314	6.309	6.308	6.308	6.308	(6.270)
	6	11.03	10.92	10.91	10.91	10.91	10.91	(/)

Note: ‘/’ denotes data not available, { } denotes results of Liew et al. [35]; ( ) denotes results of Huang et al. [20].

**Table 6:** Average differences of $\left|\frac{\Omega (\bar{M}=\bar{N}=35)-\Omega (\bar{M}=\bar{N}=15)}{\Omega (\bar{M}=\bar{N}=35)}\right|$ of the first six modes.

**Case**	**SFSF**	**SSSF**	**SCSF**	**SCSS**	**SFFF**	**SSFF**	**CSFF**	**CSSF**
Ave. Differences (%)	0.080	0.045	0.040	0.054	0.088	0.045	0.056	0.030
**Case**	**CFSF**	**CFCF**	**CSCF**	**CCFF**	**CCSF**	**CCSS**	**CCCF**	**CCCS**
Ave. Differences (%)	0.056	0.044	0.020	0.055	0.028	0.045	0.024	0.049

**Table 7:** Nondimensional natural frequencies Ω of CCCC Al/Al_2_O FGM rectangular plates.

*b*/*a*	*h*/*a*	$\bar{m}$	Mode					
			1	2	3	4	5	6
1	0.02	0	10.84	22.03	22.03	32.36	39.29	39.49
		0.5	9.184	18.67	18.67	27.44	33.32	33.48
		2	7.527	15.30	15.30	22.49	27.30	27.44
		5	7.133	14.49	14.49	21.29	25.84	25.97
	0.1	0	9.842	18.77	18.77	26.31	31.00	31.30
		0.5	8.409	16.11	16.11	22.64	26.73	26.98
		2	6.902	13.23	13.23	18.58	21.94	22.15
		5	6.451	12.27	12.27	17.15	20.18	20.38
2	0.02	0	7.413	9.593	13.48	19.05	19.23	21.34
		0.5	6.281	8.128	11.43	16.14	16.29	18.09
		2	5.148	6.662	9.363	13.23	13.35	14.83
		5	4.879	6.313	8.872	12.53	12.65	14.04
	0.1	0	6.897	8.815	12.16	16.64	16.75	18.30
		0.5	5.882	7.523	10.39	14.27	14.33	15.70
		2	4.827	6.173	8.521	11.71	11.75	12.88
		5	4.526	5.779	7.960	10.88	10.95	11.95

**Table 8:** Nondimensional natural frequencies Ω of FFFF Al/Al_2_O FGM rectangular plates.

*b*/*a*	*h*/*a*	$\bar{m}$	Mode					
			1	2	3	4	5	6
1	0.02	0	4.038	5.976	7.361	10.44	10.44	18.41
		0.5	3.421	5.067	6.238	8.849	8.849	15.59
		2	2.804	4.153	5.112	7.252	7.252	12.78
		5	2.657	3.932	4.843	6.869	6.869	12.11
	0.1	0	3.849	5.735	7.060	9.660	9.660	16.75
		0.5	3.269	4.861	5.988	8.213	8.213	14.25
		2	2.677	3.969	4.893	6.711	6.711	11.63
		5	2.525	3.752	4.621	6.312	6.312	10.91
2	0.02	0	1.656	1.997	4.389	4.511	6.690	7.604
		0.5	1.405	1.693	3.719	3.821	5.670	6.442
		2	1.152	1.387	3.048	3.132	4.647	5.280
		5	1.090	1.315	2.888	2.969	4.401	5.004
	0.1	0	1.610	1.927	4.196	4.382	6.419	7.176
		0.5	1.364	1.636	3.563	3.717	5.443	6.099
		2	1.117	1.341	2.918	3.042	4.446	4.991
		5	1.058	1.267	2.753	2.875	4.201	4.700

**Table 9:** Nondimensional natural frequencies Ω of CFFF and CFSF FGM square plates; “*” denotes the in-plane displacement-dominated mode.

BC	*h*/*a*	$\bar{m}$	Material Model	Mode					
				1	2	3	4	5	6
CFFF	0.02	0	Power-Law	1.049	2.552	6.418	8.180	9.273	16.17
		0.5		0.8888	2.162	5.437	6.930	7.857	13.70
		2		0.7284	1.772	4.456	5.678	6.439	11.23
		5		0.6907	1.680	4.224	5.383	6.102	10.63
	0.1	0	Power-Law or M-T	1.038	2.439	6.079	6.581 *	7.716	8.546
		0.5	Power-Law	0.8000	2.072	5.168	5.907 *	6.555	7.280
			M-T	0.8089	1.960	4.879	5.606 *	6.186	6.877
		2	Power-Law	0.7211	1.698	4.230	4.946 *	5.359	5.962
			M-T	0.6973	1.643	4.087	4.650 *	5.176	5.759
		5	Power-Law	0.6828	1.601	3.981	4.275 *	5.047	5.594
			M-T	0.6666	1.564	3.886	4.073 *	4.926	5.457
CFSF	0.02	0	Power-Law	4.591	6.191	11.91	14.90	16.90	23.15
		0.5		3.664	4.950	9.511	11.89	13.50	18.48
		2		3.084	4.162	8.000	10.00	11.36	15.55
		5		2.950	3.981	7.652	9.569	10.86	14.87
	0.1	0		4.401	5.820	10.89	13.45	15.05	15.57 *
		0.5		3.526	4.678	8.742	10.83	12.14	13.27 *
		2		2.963	3.922	7.316	9.071	10.16	10.99 *
		5		2.818	3.724	6.935	8.563	9.569	9.641 *

**Table 10:** Nondimensional natural frequencies Ω of FGM square plates (*h/a* = 0.1) with SSFF, CSFF, CSSF, CCFF and CCCF boundary conditions; “*” denotes the in-plane displacement-dominated mode.

BC	$\bar{m}$	Mode					
		1	2	3	4	5	6
SSFF	0	0.9943	5.011	5.600	10.63	12.16 *	14.10
	0.5	0.8432	4.254	4.754	9.044	10.92 *	12.00
	2	0.6910	3.481	3.891	7.398	9.134 *	9.806
	5	0.6538	3.286	3.672	6.951	7.896 *	9.204
CSFF	0	1.571	5.472	6.977	8.176 *	11.71	14.51
	0.5	1.333	4.648	5.936	7.340	9.984	12.35
	2	1.093	3.804	4.860	6.148 *	8.171	10.10
	5	1.033	3.585	4.569	5.313 *	7.653	9.470
CSSF	0	4.837	8.710	13.92	16.36 *	17.23	17.83
	0.5	4.113	7.416	11.89	14.63 *	14.74	15.25
	2	3.372	6.072	9.747	11.98	12.31 *	12.49
	5	3.175	5.703	9.106	10.60 *	11.23	11.64
CCFF	0	2.019	6.700	7.481	12.71	15.41 *	16.62
	0.5	1.714	5.703	6.369	10.85	13.80 *	14.22
	2	1.405	4.668	5.217	8.883	11.42 *	11.78
	5	1.327	4.385	4.900	8.300	9.983 *	10.87
CCCF	0	6.685	10.78	16.41	19.75	20.31	23.79 *
	0.5	5.701	9.207	14.01	16.90	17.43	21.36 *
	2	4.678	7.546	11.55	13.83	14.29	17.88 *
	5	4.385	7.050	10.72	12.85	13.25	15.46 *

## Appendix A

The exact closed-form solutions for vibrations of SSSS FGM rectangular plates are given herein. To satisfy SSSS boundary conditions, let

$$\begin{array}{l} u_{0}(x,y,t)=\sum_{\tilde{m}\text{=0}}^{\infty }\sum_{\tilde{n}\text{=0}}^{\infty }a_{\tilde{m}\tilde{n}}\cos(\frac{\tilde{m}\pi }{a}x)\sin(\frac{\tilde{n}\pi }{b}y)e^{i\omega t},\\ v_{0}(x,y,t)=\sum_{\tilde{m}\text{=0}}^{\infty }\sum_{\tilde{n}\text{=0}}^{\infty }b_{\tilde{m}\tilde{n}}\sin(\frac{\tilde{m}\pi }{a}x)\cos(\frac{\tilde{n}\pi }{b}y)e^{i\omega t},\\ w_{0}(x,y,t)=\sum_{\tilde{m}\text{=0}}^{\infty }\sum_{\tilde{n}\text{=0}}^{\infty }c_{\tilde{m}\tilde{n}}\sin(\frac{\tilde{m}\pi }{a}x)\sin(\frac{\tilde{n}\pi }{b}y)e^{i\omega t},\\ \psi_{x}(x,y,t)=\sum_{\tilde{m}\text{=0}}^{\infty }\sum_{\tilde{n}\text{=0}}^{\infty }d_{\tilde{m}\tilde{n}}\cos(\frac{\tilde{m}\pi }{a}x)\sin(\frac{\tilde{n}\pi }{b}y)e^{i\omega t}\\ \psi_{y}(x,y,t)=\sum_{\tilde{m}\text{=0}}^{\infty }\sum_{\tilde{n}\text{=0}}^{\infty }e_{\tilde{m}\tilde{n}}\sin(\frac{\tilde{m}\pi }{a}x)\cos(\frac{\tilde{n}\pi }{b}y)e^{i\omega t} \end{array}$$

where $a_{\tilde{m}\tilde{n}}$, $b_{\tilde{m}\tilde{n}}$, $c_{\tilde{m}\tilde{n}}$, $d_{\tilde{m}\tilde{n}}$ and $e_{\tilde{m}\tilde{n}}$ are coefficients to be determined. Substituting are coefficients to be determined. Substituting the above equations into Equations (13)–(17) yields

$$(K-\omega^{2}M)\left\{\begin{array}{c} \begin{array}{c} a_{\tilde{m}\tilde{n}}\\ b_{\tilde{m}\tilde{n}} \end{array}\\ c_{\tilde{m}\tilde{n}}\\ d_{\tilde{m}\tilde{n}}\\ e_{\tilde{m}\tilde{n}} \end{array}\right\}=0$$

where

$$\begin{array}{c} K=\left[\begin{array}{ccccc} \left(\alpha^{2}{\bar{E}}_{0}+\beta^{2}{\bar{G}}_{0}\right) & \alpha \beta ({\bar{D}}_{0}-{\bar{G}}_{0}) & 0 & \left(\alpha^{2}{\bar{E}}_{1}+\beta^{2}{\bar{G}}_{1}\right) & \alpha \beta ({\bar{D}}_{1}-{\bar{G}}_{1})\\ \alpha \beta ({\bar{D}}_{0}-{\bar{G}}_{0}) & \left(\beta^{2}{\bar{E}}_{0}+\alpha^{2}{\bar{G}}_{0}\right) & 0 & \alpha \beta ({\bar{D}}_{1}-{\bar{G}}_{1}) & \left(\beta^{2}{\bar{E}}_{1}+\alpha^{2}{\bar{G}}_{1}\right)\\ 0 & 0 & (-\alpha^{2}-\beta^{2})\kappa {\bar{G}}_{0} & \alpha \kappa {\bar{G}}_{0} & \beta \kappa {\bar{G}}_{0}\\ \left(\alpha^{2}{\bar{E}}_{1}+\beta^{2}{\bar{G}}_{1}\right) & \alpha \beta ({\bar{D}}_{1}-{\bar{G}}_{1}) & \alpha \kappa {\bar{G}}_{0} & \left(\alpha^{2}{\bar{E}}_{2}+\kappa {\bar{G}}_{0}+\beta^{2}{\bar{G}}_{2}\right) & \alpha \beta ({\bar{D}}_{2}-{\bar{G}}_{2})\\ \alpha \beta ({\bar{D}}_{1}-{\bar{G}}_{1}) & \left(\beta^{2}{\bar{E}}_{1}+\alpha^{2}{\bar{G}}_{1}\right) & \beta \kappa {\bar{G}}_{0} & \alpha \beta ({\bar{D}}_{2}-{\bar{G}}_{2}) & \left(\beta^{2}{\bar{E}}_{2}+\kappa {\bar{G}}_{0}+\alpha^{2}{\bar{G}}_{2}\right) \end{array}\right],\;\\ M=\left[\begin{array}{ccccc} I_{0} & 0 & 0 & I_{1} & 0\\ 0 & I_{0} & 0 & 0 & I_{1}\\ 0 & 0 & I_{0} & 0 & 0\\ I_{1} & 0 & 0 & I_{2} & 0\\ 0 & I_{1} & 0 & 0 & I_{2} \end{array}\right],\;\alpha =\frac{\tilde{m}\pi }{a}\;\mathrm{and}\;\beta =\frac{\tilde{n}\pi }{b}. \end{array}$$
